# Supplementary material for: Muscle cell-type diversification is driven by bHLH transcription factor expansion and extensive effector gene duplications
Source: Nat Commun. 2023 Mar 29;14:1747. doi: 10.1038/s41467-023-37220-6 (PMC10060217; doi:10.1038/s41467-023-37220-6)
Supplement: Supplementary file 1 — Supplementary Information [file 41467_2023_37220_MOESM1_ESM.pdf]

Title: Muscle cell-type diversification is driven by bHLH transcription factor expansion and extensive effector gene duplications in a sea anemone

Authors: Alison G. Cole[1,2 *†], Stefan M. Jahnel[1,3†], Sabrina Kaul[1], Julia Steger[1], Julia Hagauer[1], Andreas Denner[1], Patricio Ferrer Murguia[1], Elisabeth Taudes[1], Bob Zimmermann[1], Robert Reischl[1], Patrick Steinmetz[1,4], Ulrich Technau[1,2,5 *]

SUPPLEMENTARY INFORMATION (this document)

Supplementary Figures 1:10

Supplementary Figure 1

a Dissection procedure. First, tentacles were cut off close to the tentacle base, indicated with the dashed line at the very top (e). Then all mesenteries were removed in order to expose the body wall. A small piece of a single mesentery, outlined by dashed line (c) was collected for dissociation. The pharynx was isolated and longitudinally cut in two halves. Only one half was used for library preparation (d). Finally, a rectangular piece of tissue was excised from the body wall, the inset shows its outline. (b). All scale bars 500 µm. **b** Body wall. Inset shows the outline of the tissue piece. The horizontal lines correspond to the parietal muscle. The inter-parietal bodywall (BW_interP) sample was isolated from between these ridges. **c** Mesentery. The retractor muscle is the brown tissue in the center. **d** Pharynx. The pharynx+bodywall (pharynx2) sample did not have the overlaying epithelial layers removed. **e** Tentacle. **f** Schematic representation of transcriptome generation. Isolated tissues described above were dissociated and processed with a 10xGenomics Chromium single cell controller (top). To generate the bulk transcriptomes, the mesentery was first removed from an MyHC-st::mCherry transgenic animal, and the central retractor-muscle containing piece was dissociated. Cell suspensions were FACS sorted into fluorescent (muscle: red) and non-fluorescent (non-muscle:white) fractions. RNAseq libraries were generated from each isolated cell fraction in bulk. **g** Cells from each library show similar recovered numbers of genes detected (nFeature_RNA), transcripts (nCount_RNA) and mitochondrial fraction (percent.mt). Colors indicate the separate libraries as in (f: upper panel).

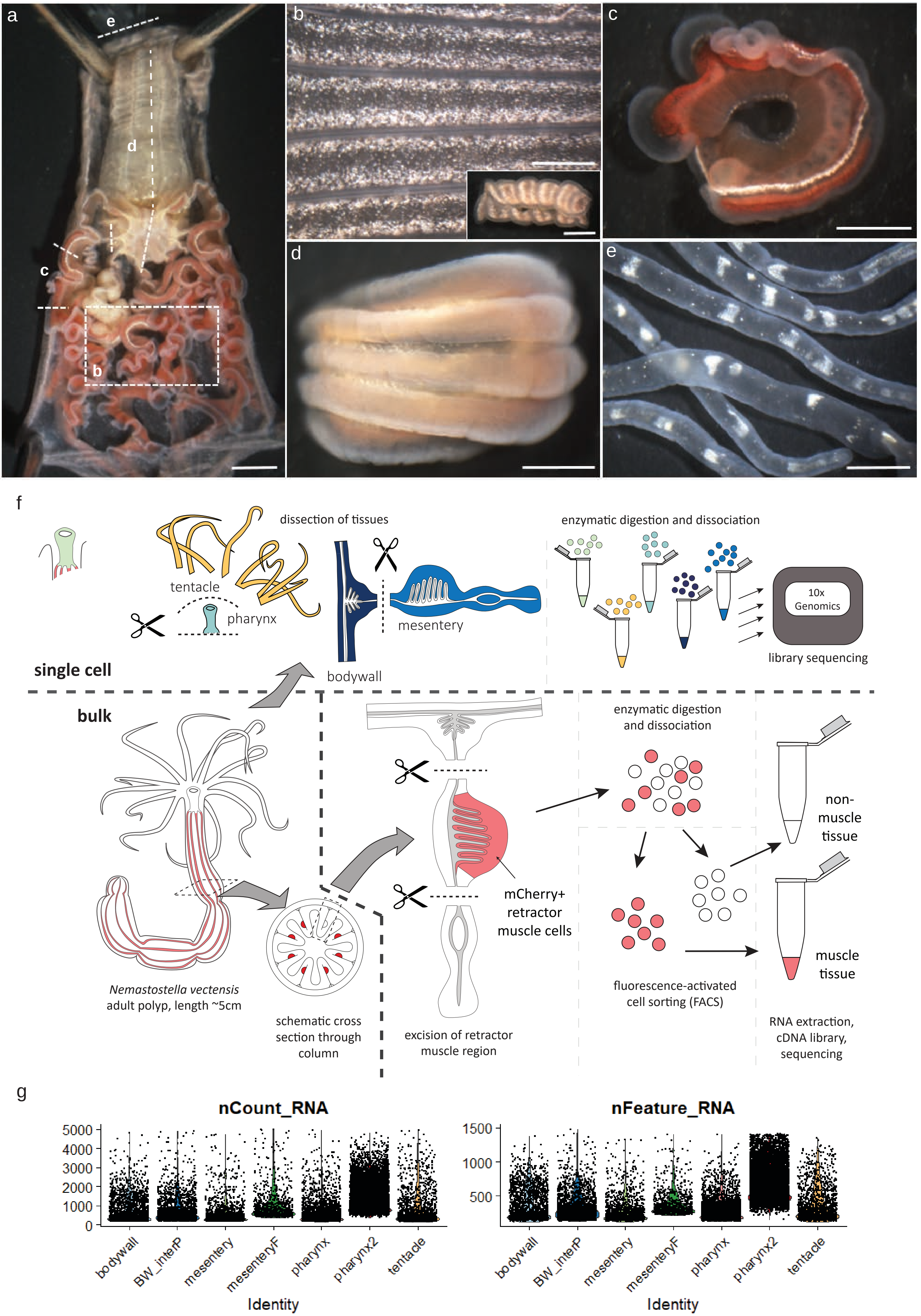
a
b
c
d
e
f
dissection of tissues
tentacle
pharynx
mesentery
bodywall
enzymatic digestion and dissociation
10x Genomics
library sequencing
single cell
bulk
Nemastostella vectensis
adult polyp, length ~5cm
schematic cross section through column
excision of retractor muscle region
mCherry+ retractor muscle cells
enzymatic digestion and dissociation
fluorescence-activated cell sorting (FACS)
non-muscle tissue
muscle tissue
RNA extraction, cDNA library, sequencing
g
nCount_RNA
nFeature_RNA
5000
4000
3000
2000
1000
0
1500
1000
500
bodywall
BW_interP
mesentery
mesenteryF
pharynx
pharynx2
tentacle
Identity

Supplementary Figure 2

Processing of the tissue dataset with the Seurat Vs3 package in R. **a,b** UMAP dimensional reduction cell plot colored by labeled cell clusters (a), or library of origin (b). **c** DotPlot of the expression profile of the top three differentially expressed genes for each cluster of cells. **d** Expression profile of the set of the genes upregulated in the BULK retractor muscle dataset that are represented by at least 50 reads in the single cell dataset, plotted as a DotPlot on the single cell dataset. This gene set clearly identifies a retractor muscle cell cluster. See also **Supplementary Data 1.S1** for full gene lists.

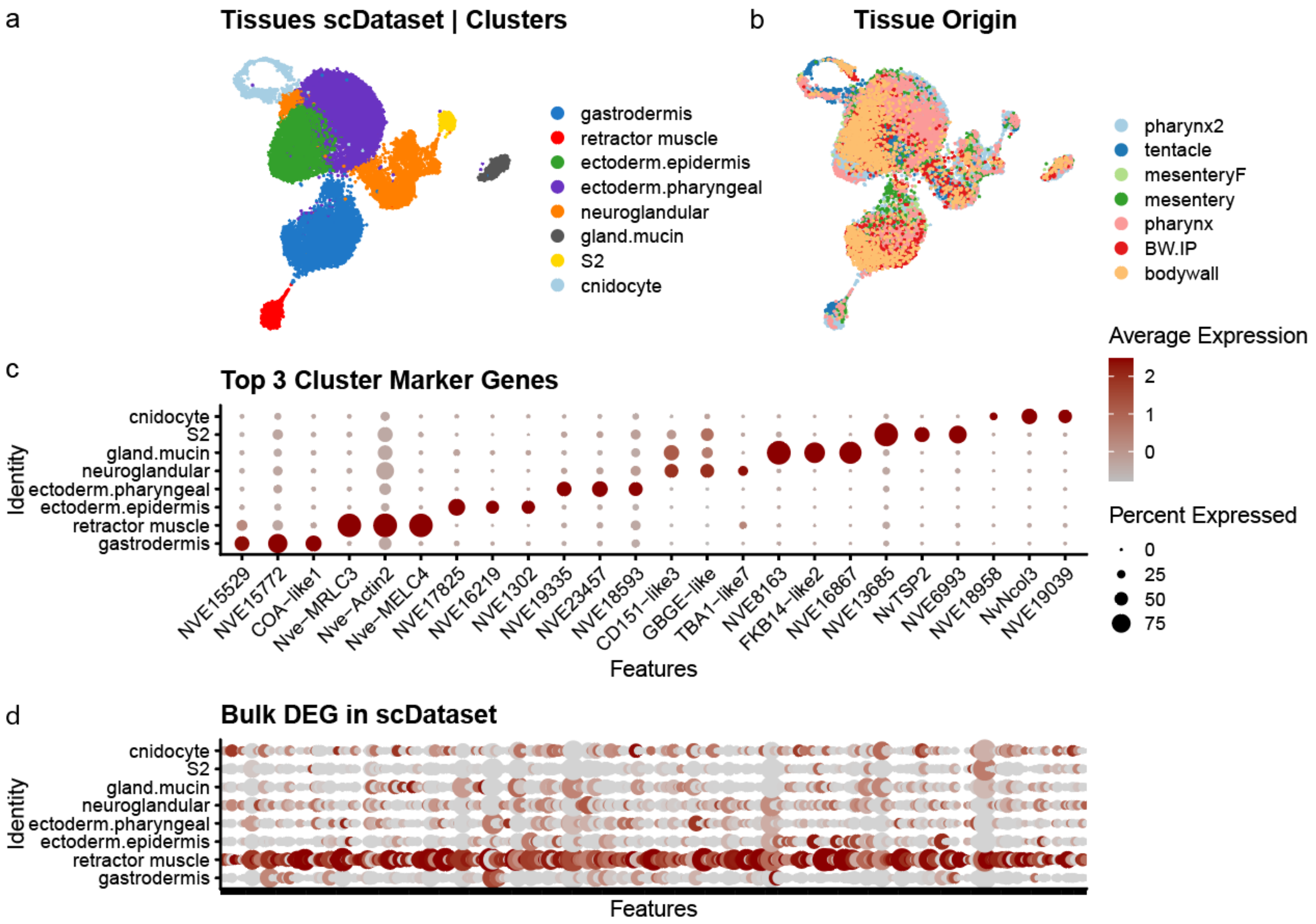
a
Tissues scDataset | Clusters
gastrodermis
retractor muscle
ectoderm.epidermis
ectoderm.pharyngeal
neuroglandular
gland.mucin
S2
cnidocyte
b
Tissue Origin
pharynx2
tentacle
mesenteryF
mesentery
pharynx
BW.IP
bodywall
c
Top 3 Cluster Marker Genes
Average Expression
2
1
0
Percent Expressed
0
25
50
75
Identity
Features
NVE15529
NVE15772
COA-like1
Nve-MRLC3
Nve-Actin2
Nve-MELC4
NVE17825
NVE16219
NVE1302
NVE19335
NVE23457
NVE18593
CD151-like3
GBGE-like
TBA1-like7
NVE8163
FKB14-like2
NVE16867
NVE13685
NvTSP2
NVE6993
NVE18958
NvNcol3
NVE19039
d
Bulk DEG in scDataset
Identity
Features

Supplementary Figure 3

Processing of the muscle/endodermal subset. **a** Distribution of cells from separate libraries plotted onto the UMAP dimensional reduction cell plot demonstrates clear oral polarity (pharynx: red/pink). Cluster labelling is as in **Fig. 1e**. **b** The portion of each library assigned to the different clusters is represented as a bar plot. Yellow highlights indicate unexpected contributions of cells, likely due to inherently fuzzy cluster boundaries. Note that cells of the TR (blue) are restricted to the tentacle and pharyngeal libraries, and the parietal muscle (dark green) is restricted to the bodywall library. The circular muscle (light green) is distributed across all libraries, whereas the MR (dark red) is derived primarily from the mesentery and pharyngeal libraries. There are some cells of MR identity also from the tentacle library that most likely represents mis-clustering due to convergent transcriptomes in the most-differentiated retractor muscle cells. No cells of the inter-muscular membrane (ImM) from the BW-interP nor tentacle libraries, as would be expected for these cell types. **c** Expression of the myosin heavy chain – striated type (myHC-st) across the subset; note the distinct absence of expression (grey) in the ImM and non-muscle gastrodermis. **d** GO-term analysis of differentially expressed genes from each cluster. Terms related to muscle and/or actin processing are highlighted in dark red. Note the absence of these terms in the other clusters. **e,f** Dotplot illustrating the top 10 most significantly expressed genes from the FindAllMarkers function for (e) structural and (f) regulatory gene sets, using the Wilcoxon Rank Sum test and filtering for only genes with p.value < 0.001 returned. **f** A rich regulatory molecule profile of genes previously demonstrated to express in the pharyngeal and oral territories helps identify gastrodermal cells from the oral (endomes.oral) and pharyngeal/septal filament territories (endomes.phary), including for example *twist, lbx, moxB,D*[9,24]; **see also Supplementary Data 1.S2**.

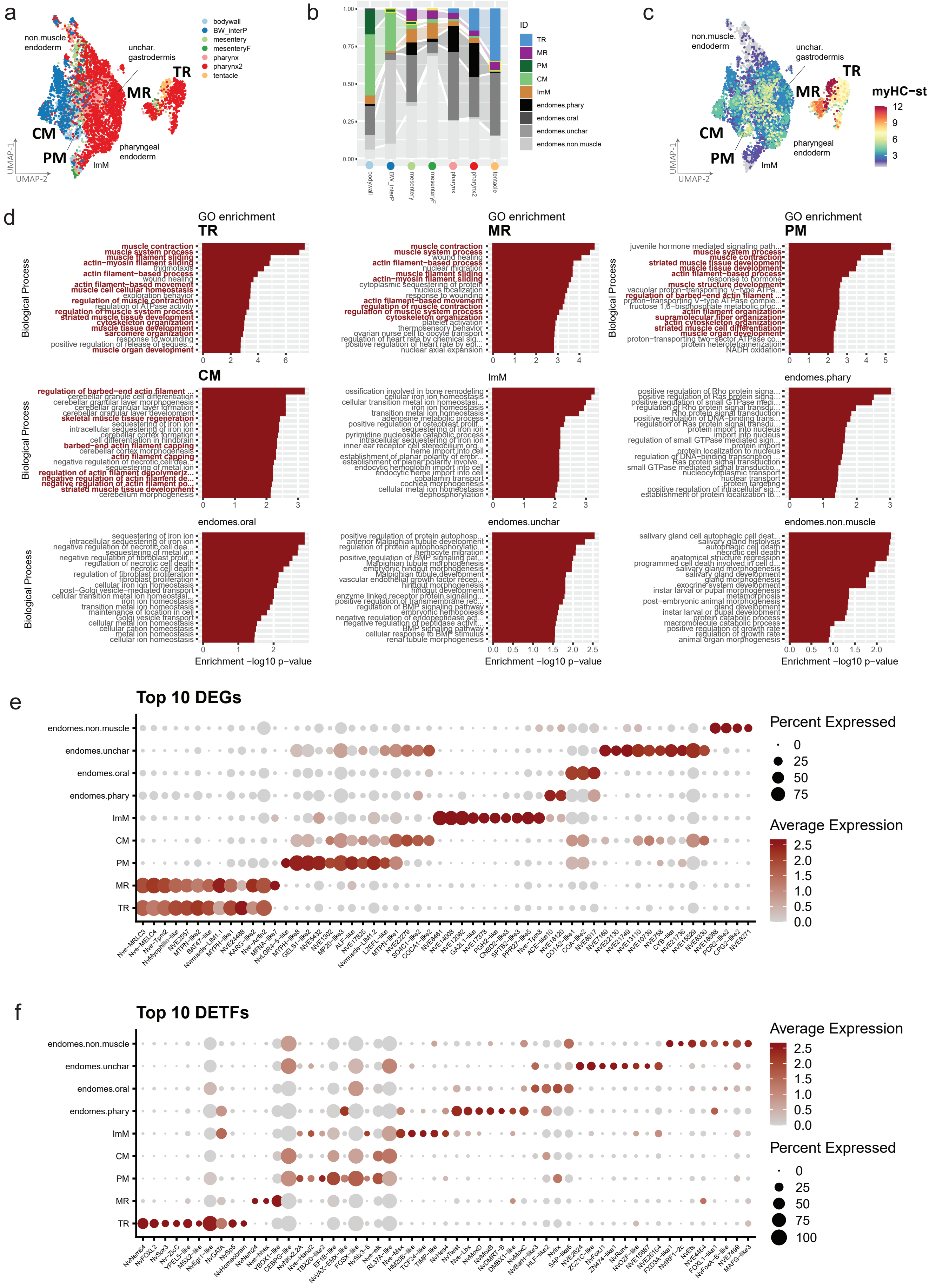

a
non.muscle endoderm
unchar. gastrodermis
TR
MR
CM
PM
ImM
pharyngeal endoderm
UMAP-1
UMAP-2
bodywall
BW_interP
mesentery
mesenteryF
pharynx
pharynx2
tentacle
b
ID
TR
MR
PM
CM
ImM
endomes.phary
endomes.oral
endomes.unchar
endomes.non.muscle
c
myHC-st
d
GO enrichment
TR
MR
PM
CM
ImM
endomes.phary
endomes.oral
endomes.unchar
endomes.non.muscle
Biological Process
Enrichment −log10 p−value
muscle contraction
muscle system process
muscle filament sliding
actin−myosin filament sliding
actin filament−based process
actin filament−based movement
muscle cell cellular homeostasis
regulation of muscle contraction
regulation of muscle system process
striated muscle tissue development
cytoskeleton organization
muscle tissue development
sarcomere organization
muscle organ development
e
Top 10 DEGs
Percent Expressed
Average Expression
f
Top 10 DETFs
Average Expression
Percent Expressed

Supplementary Figure 4

Spatial expression profiles for selected structural genes by colorimetric *in situ* hybridization. Genes representative of the bodywall (slow) gene set are shown on the left. The distribution of each gene within the scRNA dataset is shown in the center. Two genes (yellow bars) are paralogs NOT expressed within differentiated muscle tissue. Genes representative of the fast-contracting retractor muscle gene set are shown in the right panel. In all cases the spatial restriction to the retractor muscles as indicated by the single cell data is confirmed. Scale bars are 50μM.

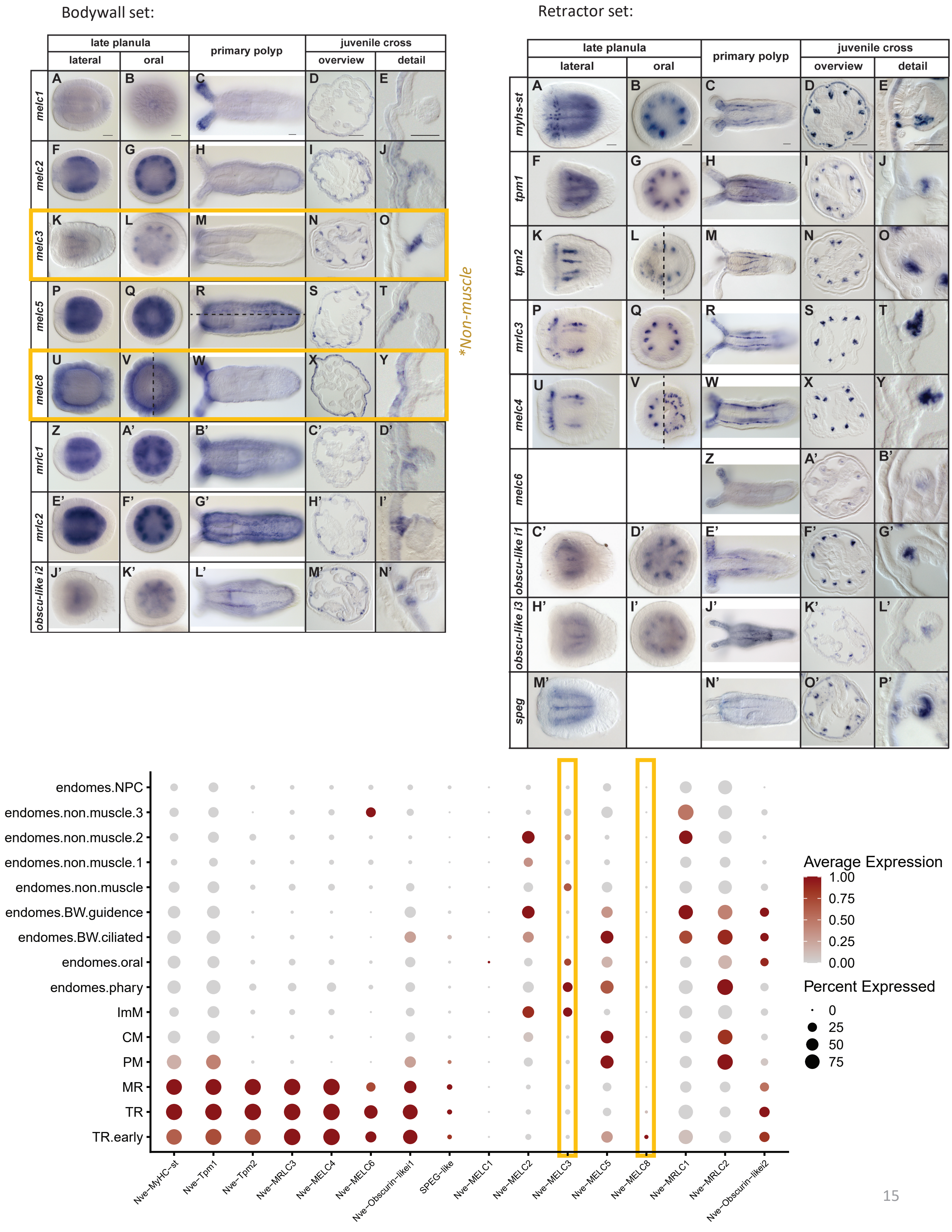
Bodywall set:
late planula
lateral
oral
primary polyp
juvenile cross
overview
detail
melc1
melc2
melc3
melc5
melc8
mrlc1
mrlc2
obscu-like i2
*Non-muscle
Retractor set:
myhs-st
tpm1
tpm2
mrlc3
melc4
melc6
obscu-like i1
obscu-like i3
speg
endomes.NPC
endomes.non.muscle.3
endomes.non.muscle.2
endomes.non.muscle.1
endomes.non.muscle
endomes.BW.guidence
endomes.BW.ciliated
endomes.oral
endomes.phary
ImM
CM
PM
MR
TR
TR.early
Nve−MyHC−st
Nve−Tpm1
Nve−Tpm2
Nve−MRLC3
Nve−MELC4
Nve−MELC6
Nve−Obscurin−likei1
SPEG−like
Nve−MELC1
Nve−MELC2
Nve−MELC3
Nve−MELC5
Nve−MELC8
Nve−MRLC1
Nve−MRLC2
Nve−Obscurin−likei2
Average Expression
1.00
0.75
0.50
0.25
0.00
Percent Expressed
0
25
50
75

Supplementary Figure 5

Set of all genes expressed within any of the differentiated muscle cell populations, but largely absent from the remaining ectodermal cells of the full dataset. Genes were selected as having at least 10 reads associated with any muscle cell cluster from the data subset, but less than 200 reads from the remaining ectodermally-derived dataset. **a** Many genes of the slow muscle group show extensive expression also within the other non-muscle endodermal cell clusters. **b** The VennDiagram shows the overlapping components, whose expression profiles are illustrated as DotPlots on the full dataset. **c** The set of genes common to both retractor muscles (tentacle: TR and mesentery: MR).

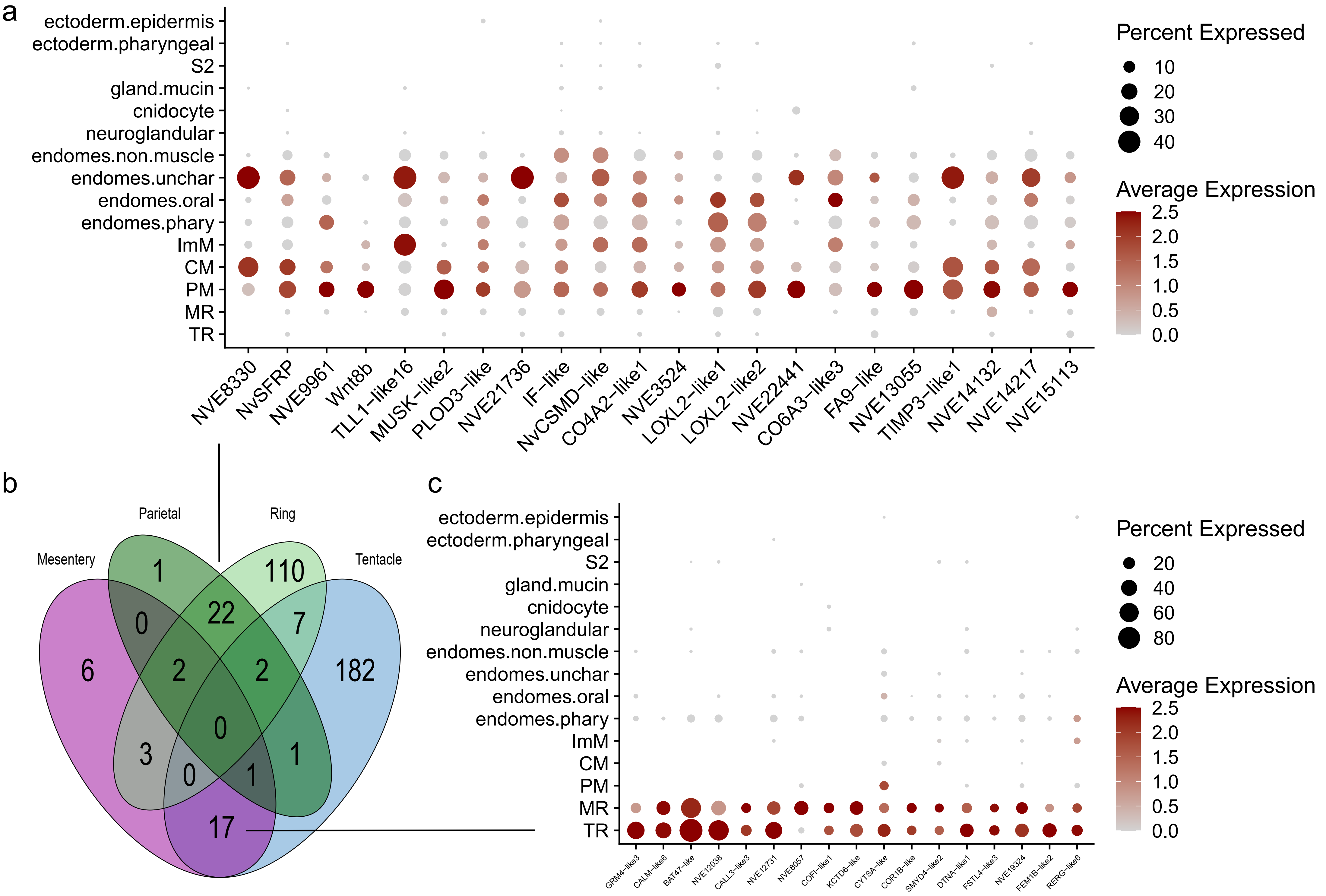

a
ectoderm.epidermis
ectoderm.pharyngeal
S2
gland.mucin
cnidocyte
neuroglandular
endomes.non.muscle
endomes.unchar
endomes.oral
endomes.phary
ImM
CM
PM
MR
TR
NVE8330
NvSFRP
NVE9961
Wnt8b
TLL1−like16
MUSK−like2
PLOD3−like
NVE21736
IF−like
NvCSMD−like
CO4A2−like1
NVE3524
LOXL2−like1
LOXL2−like2
NVE22441
CO6A3−like3
FA9−like
NVE13055
TIMP3−like1
NVE14132
NVE14217
NVE15113
Percent Expressed
10
20
30
40
Average Expression
2.5
2.0
1.5
1.0
0.5
0.0
b
Parietal
Ring
Mesentery
Tentacle
1
110
0
22
7
6
2
2
182
0
3
0
1
1
17
c
ectoderm.epidermis
ectoderm.pharyngeal
S2
gland.mucin
cnidocyte
neuroglandular
endomes.non.muscle
endomes.unchar
endomes.oral
endomes.phary
ImM
CM
PM
MR
TR
GRM4−like3
CALM−like6
BAT47−like
NVE12038
CALL3−like3
NVE12731
NVE8057
COFI−like1
KCTD6−like
CYTSA−like
COR1B−like
SMYD4−like2
DTNA−like1
FSTL4−like3
NVE19324
FEM1B−like2
RERG−like6
Percent Expressed
20
40
60
80
Average Expression
2.5
2.0
1.5
1.0
0.5
0.0

Supplementary Figure 6

Gene trees for paralogous structural genes Expression profiles in terms of muscle type are indicated as colours (Fast muscle: orange; Slow muscle: green). All *Nematostella* gene models are highlighted in red. Colored boxes along the right side indicate the distribution of cnidarian (bright green) vs. bilaterian (cyan) sequences. Nodes are labeled with SH-aLRT /ultrafast bootstrap support (%). **A** Calmodulin **B** Actin **C** MELC **D** Calponin **E** MRLC **F** Tropomyosin. *Aqu: Amphimedon queenslandica, Adi: Acropora digitifera, Hvu: Hydra vulgaris, Epa: Exaiptasia pallida, Cte: Capitella teleta, Dme: Drosophila melanogaster, Che: Clytia hemisphaerica, Mmu: Mus musculus, ga: Gallus gallus*, *Xtr: Xenopus tropicalis, Hsa: Homo sapiens, Dre: Danio rerio*

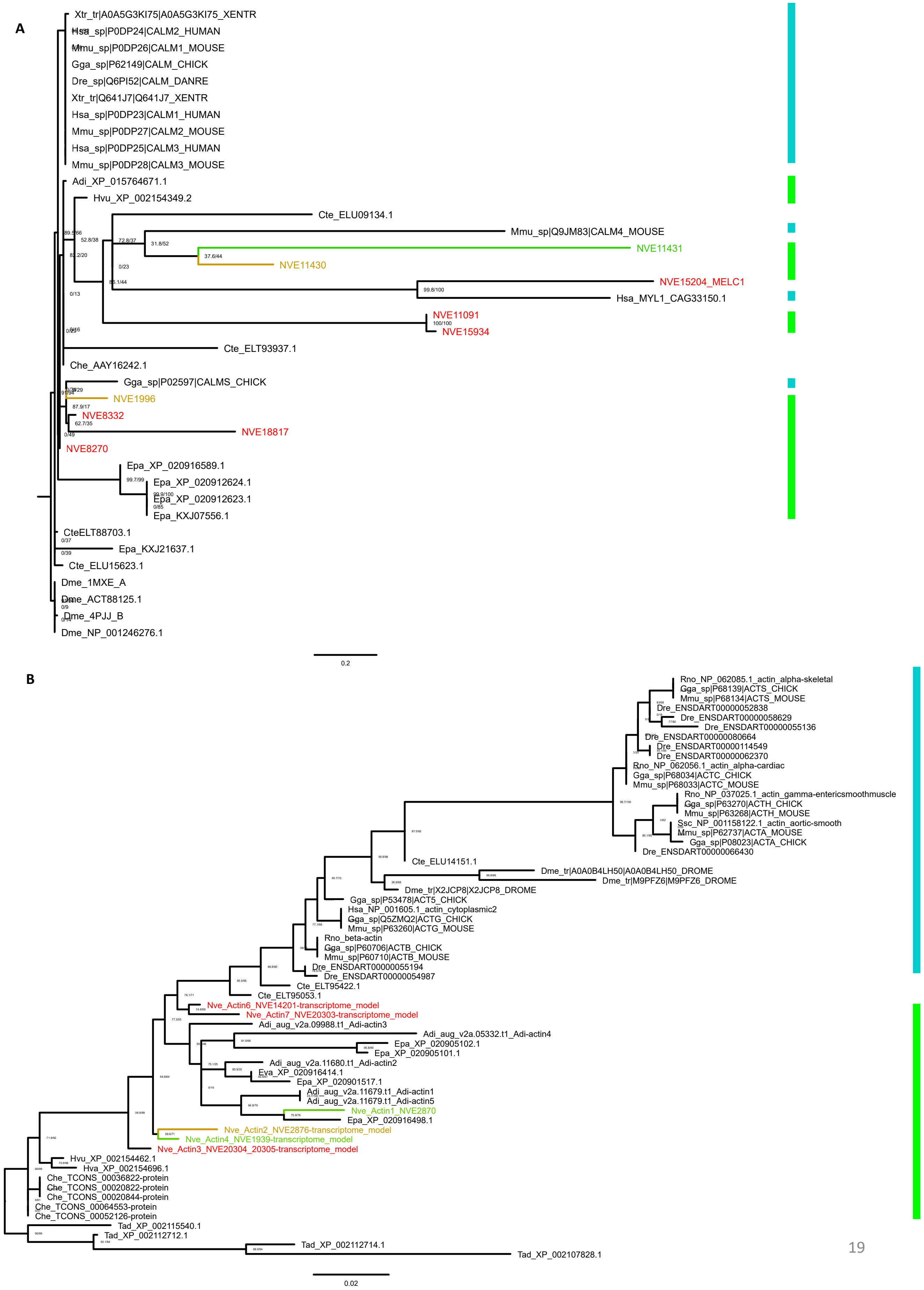
A
Xtr_tr|A0A5G3KI75|A0A5G3KI75_XENTR
Hsa_sp|P0DP24|CALM2_HUMAN
Mmu_sp|P0DP26|CALM1_MOUSE
Gga_sp|P62149|CALM_CHICK
Dre_sp|Q6PI52|CALM_DANRE
Xtr_tr|Q641J7|Q641J7_XENTR
Hsa_sp|P0DP23|CALM1_HUMAN
Mmu_sp|P0DP27|CALM2_MOUSE
Hsa_sp|P0DP25|CALM3_HUMAN
Mmu_sp|P0DP28|CALM3_MOUSE
Adi_XP_015764671.1
Hvu_XP_002154349.2
Cte_ELU09134.1
Mmu_sp|Q9JM83|CALM4_MOUSE
NVE11431
NVE11430
NVE15204_MELC1
Hsa_MYL1_CAG33150.1
NVE11091
NVE15934
Cte_ELT93937.1
Che_AAY16242.1
Gga_sp|P02597|CALMS_CHICK
NVE1996
NVE8332
NVE18817
NVE8270
Epa_XP_020916589.1
Epa_XP_020912624.1
Epa_XP_020912623.1
Epa_KXJ07556.1
CteELT88703.1
Epa_KXJ21637.1
Cte_ELU15623.1
Dme_1MXE_A
Dme_ACT88125.1
Dme_4PJJ_B
Dme_NP_001246276.1
0.2
B
Rno_NP_062085.1_actin_alpha-skeletal
Gga_sp|P68139|ACTS_CHICK
Mmu_sp|P68134|ACTS_MOUSE
Dre_ENSDART00000052838
Dre_ENSDART00000058629
Dre_ENSDART00000055136
Dre_ENSDART00000080664
Dre_ENSDART00000114549
Dre_ENSDART00000062370
Rno_NP_062056.1_actin_alpha-cardiac
Gga_sp|P68034|ACTC_CHICK
Mmu_sp|P68033|ACTC_MOUSE
Rno_NP_037025.1_actin_gamma-entericsmoothmuscle
Gga_sp|P63270|ACTH_CHICK
Mmu_sp|P63268|ACTH_MOUSE
Ssc_NP_001158122.1_actin_aortic-smooth
Mmu_sp|P62737|ACTA_MOUSE
Gga_sp|P08023|ACTA_CHICK
Dre_ENSDART00000066430
Cte_ELU14151.1
Dme_tr|A0A0B4LH50|A0A0B4LH50_DROME
Dme_tr|M9PFZ6|M9PFZ6_DROME
Dme_tr|X2JCP8|X2JCP8_DROME
Gga_sp|P53478|ACT5_CHICK
Hsa_NP_001605.1_actin_cytoplasmic2
Gga_sp|Q5ZMQ2|ACTG_CHICK
Mmu_sp|P63260|ACTG_MOUSE
Rno_beta-actin
Gga_sp|P60706|ACTB_CHICK
Mmu_sp|P60710|ACTB_MOUSE
Dre_ENSDART00000055194
Dre_ENSDART00000054987
Cte_ELT95422.1
Cte_ELT95053.1
Nve_Actin6_NVE14201-transcriptome_model
Nve_Actin7_NVE20303-transcriptome_model
Adi_aug_v2a.09988.t1_Adi-actin3
Adi_aug_v2a.05332.t1_Adi-actin4
Epa_XP_020905102.1
Epa_XP_020905101.1
Adi_aug_v2a.11680.t1_Adi-actin2
Epa_XP_020916414.1
Epa_XP_020901517.1
Adi_aug_v2a.11679.t1_Adi-actin1
Adi_aug_v2a.11679.t1_Adi-actin5
Nve_Actin1_NVE2870
Epa_XP_020916498.1
Nve_Actin2_NVE2876-transcriptome_model
Nve_Actin4_NVE1939-transcriptome_model
Nve_Actin3_NVE20304_20305-transcriptome_model
Hvu_XP_002154462.1
Hva_XP_002154696.1
Che_TCONS_00036822-protein
Che_TCONS_00020822-protein
Che_TCONS_00020844-protein
Che_TCONS_00064553-protein
Che_TCONS_00052126-protein
Tad_XP_002115540.1
Tad_XP_002112712.1
Tad_XP_002112714.1
Tad_XP_002107828.1
0.02

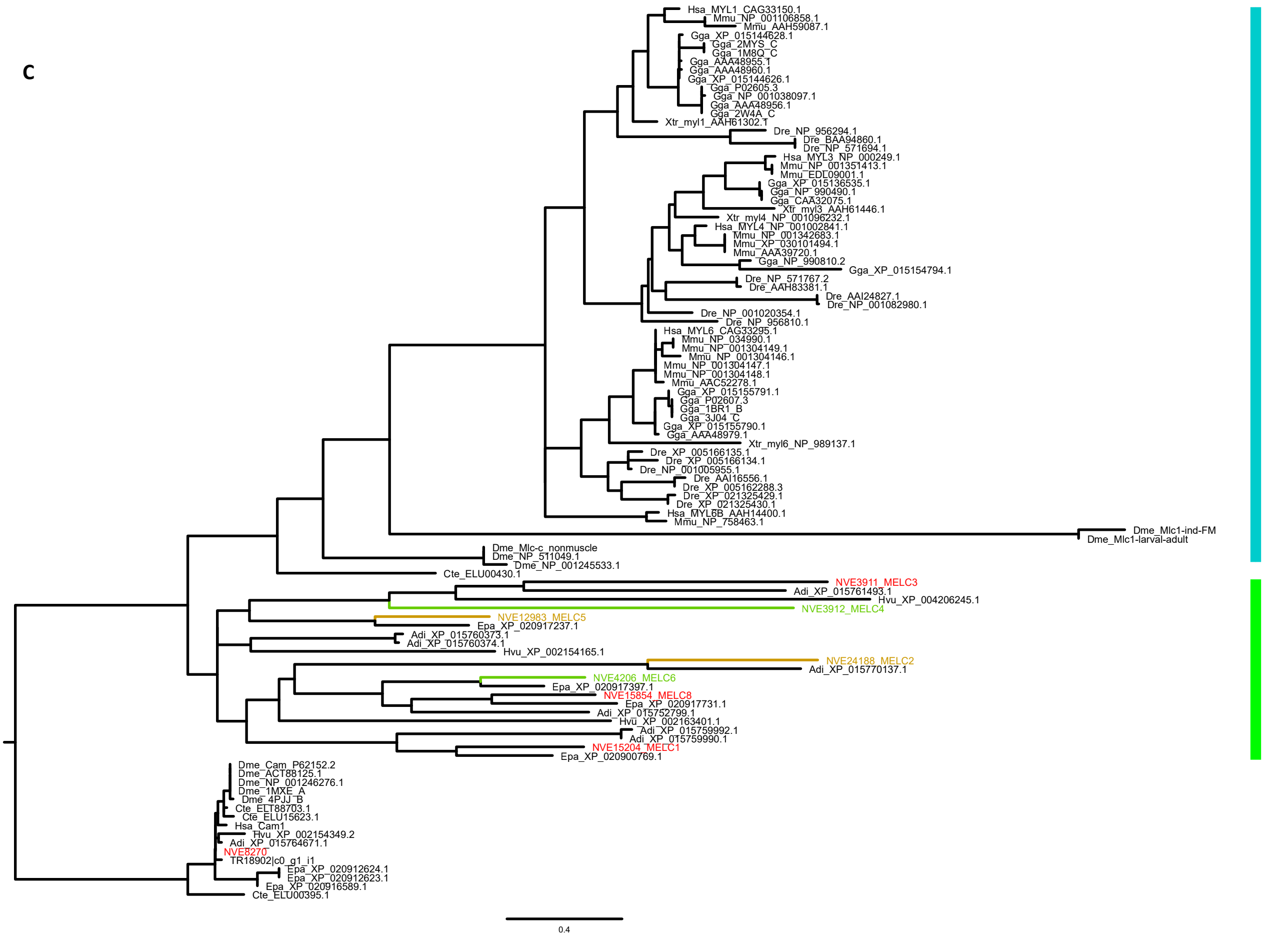
C
Hsa_MYL1_CAG33150.1
Mmu_NP_001106858.1
Mmu_AAH59087.1
Gga_XP_015144628.1
Gga_2MYS_C
Gga_1M8Q_C
Gga_AAA48955.1
Gga_AAA48960.1
Gga_XP_015144626.1
Gga_P02605.3
Gga_NP_001038097.1
Gga_AAA48956.1
Gga_2W4A_C
Xtr_myl1_AAH61302.1
Dre_NP_956294.1
Dre_BAA94860.1
Dre_NP_571694.1
Hsa_MYL3_NP_000249.1
Mmu_NP_001351413.1
Mmu_EDL09001.1
Gga_XP_015136535.1
Gga_NP_990490.1
Gga_CAA32075.1
Xtr_myl3_AAH61446.1
Xtr_myl4_NP_001096232.1
Hsa_MYL4_NP_001002841.1
Mmu_NP_001342683.1
Mmu_XP_030101494.1
Mmu_AAA39720.1
Gga_NP_990810.2
Gga_XP_015154794.1
Dre_NP_571767.2
Dre_AAH83381.1
Dre_AAI24827.1
Dre_NP_001082980.1
Dre_NP_001020354.1
Dre_NP_956810.1
Hsa_MYL6_CAG33295.1
Mmu_NP_034990.1
Mmu_NP_001304149.1
Mmu_NP_001304146.1
Mmu_NP_001304147.1
Mmu_NP_001304148.1
Mmu_AAC52278.1
Gga_XP_015155791.1
Gga_P02607.3
Gga_1BR1_B
Gga_3J04_C
Gga_XP_015155790.1
Gga_AAA48979.1
Xtr_myl6_NP_989137.1
Dre_XP_005166135.1
Dre_XP_005166134.1
Dre_NP_001005955.1
Dre_AAI16556.1
Dre_XP_005162288.3
Dre_XP_021325429.1
Dre_XP_021325430.1
Hsa_MYL6B_AAH14400.1
Mmu_NP_758463.1
Dme_Mlc1-ind-FM
Dme_Mlc1-larval-adult
Dme_Mlc-c_nonmuscle
Dme_NP_511049.1
Dme_NP_001245533.1
Cte_ELU00430.1
NVE3911_MELC3
Adi_XP_015761493.1
Hvu_XP_004206245.1
NVE3912_MELC4
NVE12983_MELC5
Epa_XP_020917237.1
Adi_XP_015760373.1
Adi_XP_015760374.1
Hvu_XP_002154165.1
NVE24188_MELC2
Adi_XP_015770137.1
NVE4206_MELC6
Epa_XP_020917397.1
NVE15854_MELC8
Epa_XP_020917731.1
Adi_XP_015752799.1
Hvu_XP_002163401.1
Adi_XP_015759992.1
Adi_XP_015759990.1
NVE15204_MELC1
Epa_XP_020900769.1
Dme_Cam_P62152.2
Dme_ACT88125.1
Dme_NP_001246276.1
Dme_1MXE_A
Dme_4PJJ_B
Cte_ELT88703.1
Cte_ELU15623.1
Hsa_Cam1
Hvu_XP_002154349.2
Adi_XP_015764671.1
NVE8270
TR18902|c0_g1_i1
Epa_XP_020912624.1
Epa_XP_020912623.1
Epa_XP_020916589.1
Cte_ELU00395.1
0.4

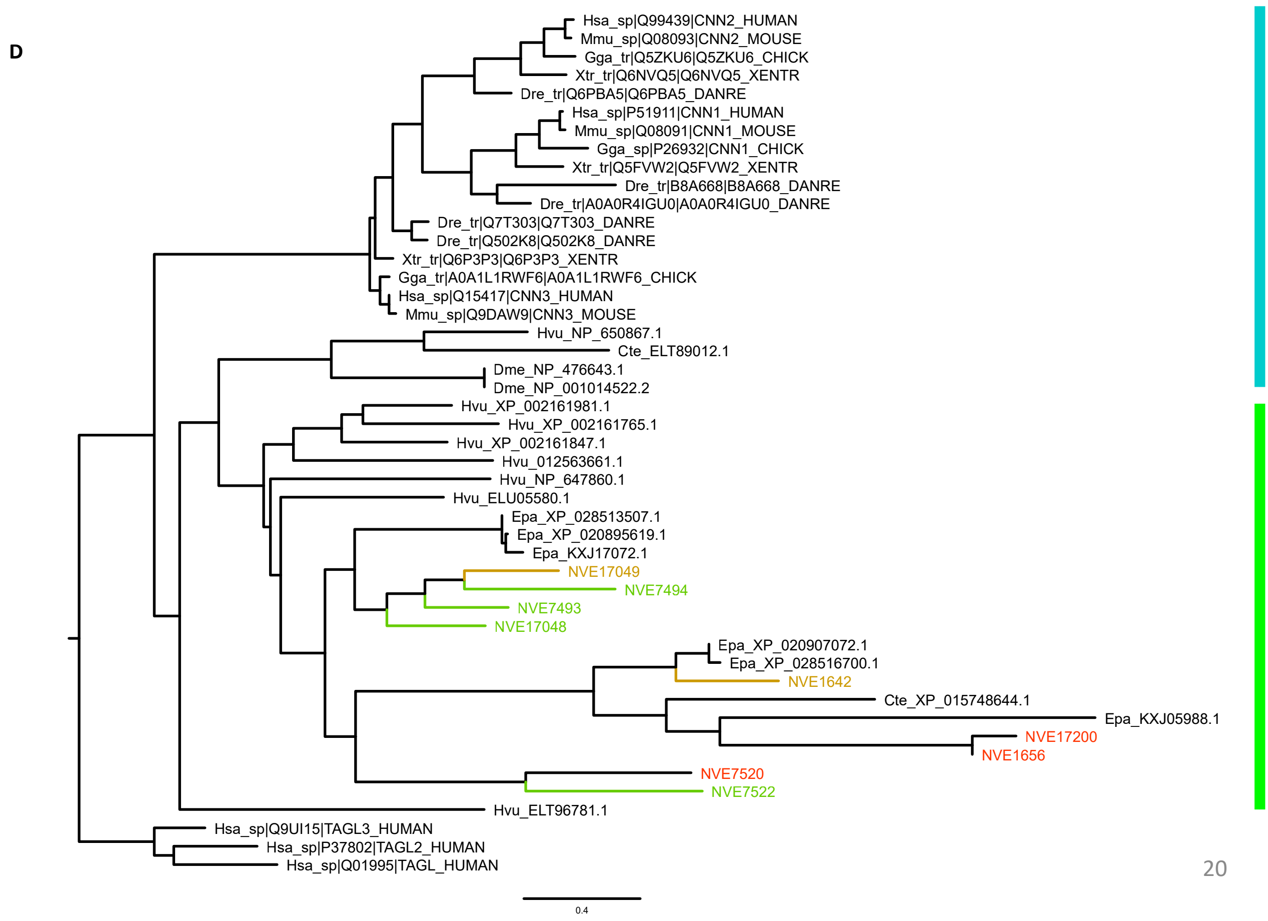
D
Hsa_sp|Q99439|CNN2_HUMAN
Mmu_sp|Q08093|CNN2_MOUSE
Gga_tr|Q5ZKU6|Q5ZKU6_CHICK
Xtr_tr|Q6NVQ5|Q6NVQ5_XENTR
Dre_tr|Q6PBA5|Q6PBA5_DANRE
Hsa_sp|P51911|CNN1_HUMAN
Mmu_sp|Q08091|CNN1_MOUSE
Gga_sp|P26932|CNN1_CHICK
Xtr_tr|Q5FVW2|Q5FVW2_XENTR
Dre_tr|B8A668|B8A668_DANRE
Dre_tr|A0A0R4IGU0|A0A0R4IGU0_DANRE
Dre_tr|Q7T303|Q7T303_DANRE
Dre_tr|Q502K8|Q502K8_DANRE
Xtr_tr|Q6P3P3|Q6P3P3_XENTR
Gga_tr|A0A1L1RWF6|A0A1L1RWF6_CHICK
Hsa_sp|Q15417|CNN3_HUMAN
Mmu_sp|Q9DAW9|CNN3_MOUSE
Hvu_NP_650867.1
Cte_ELT89012.1
Dme_NP_476643.1
Dme_NP_001014522.2
Hvu_XP_002161981.1
Hvu_XP_002161765.1
Hvu_XP_002161847.1
Hvu_012563661.1
Hvu_NP_647860.1
Hvu_ELU05580.1
Epa_XP_028513507.1
Epa_XP_020895619.1
Epa_KXJ17072.1
NVE17049
NVE7494
NVE7493
NVE17048
Epa_XP_020907072.1
Epa_XP_028516700.1
NVE1642
Cte_XP_015748644.1
Epa_KXJ05988.1
NVE17200
NVE1656
NVE7520
NVE7522
Hvu_ELT96781.1
Hsa_sp|Q9UI15|TAGL3_HUMAN
Hsa_sp|P37802|TAGL2_HUMAN
Hsa_sp|Q01995|TAGL_HUMAN
0.4

E

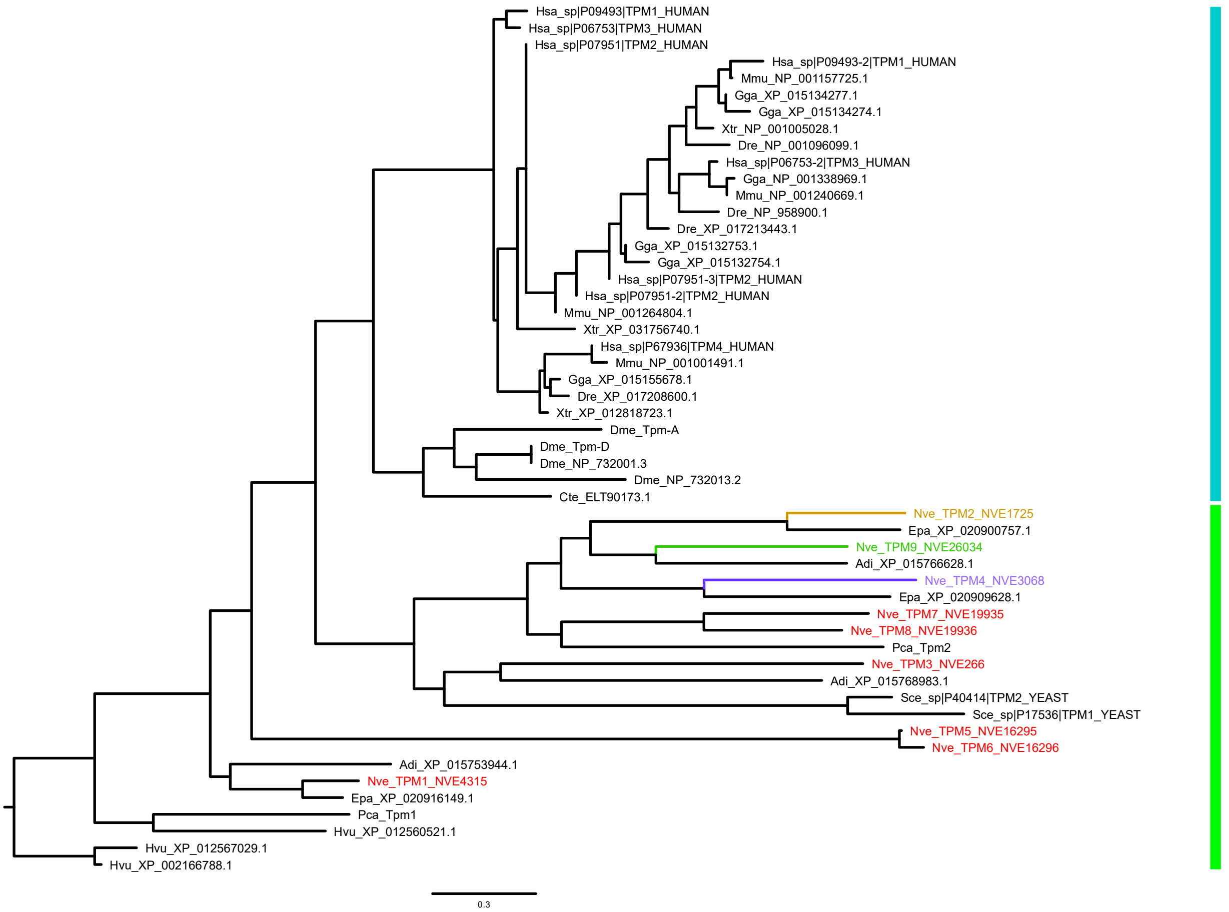

Hsa_sp|P09493|TPM1_HUMAN
Hsa_sp|P06753|TPM3_HUMAN
Hsa_sp|P07951|TPM2_HUMAN
Hsa_sp|P09493-2|TPM1_HUMAN
Mmu_NP_001157725.1
Gga_XP_015134277.1
Gga_XP_015134274.1
Xtr_NP_001005028.1
Dre_NP_001096099.1
Hsa_sp|P06753-2|TPM3_HUMAN
Gga_NP_001338969.1
Mmu_NP_001240669.1
Dre_NP_958900.1
Dre_XP_017213443.1
Gga_XP_015132753.1
Gga_XP_015132754.1
Hsa_sp|P07951-3|TPM2_HUMAN
Hsa_sp|P07951-2|TPM2_HUMAN
Mmu_NP_001264804.1
Xtr_XP_031756740.1
Hsa_sp|P67936|TPM4_HUMAN
Mmu_NP_001001491.1
Gga_XP_015155678.1
Dre_XP_017208600.1
Xtr_XP_012818723.1
Dme_Tpm-A
Dme_Tpm-D
Dme_NP_732001.3
Dme_NP_732013.2
Cte_ELT90173.1
Nve_TPM2_NVE1725
Epa_XP_020900757.1
Nve_TPM9_NVE26034
Adi_XP_015766628.1
Nve_TPM4_NVE3068
Epa_XP_020909628.1
Nve_TPM7_NVE19935
Nve_TPM8_NVE19936
Pca_Tpm2
Nve_TPM3_NVE266
Adi_XP_015768983.1
Sce_sp|P40414|TPM2_YEAST
Sce_sp|P17536|TPM1_YEAST
Nve_TPM5_NVE16295
Nve_TPM6_NVE16296
Adi_XP_015753944.1
Nve_TPM1_NVE4315
Epa_XP_020916149.1
Pca_Tpm1
Hvu_XP_012560521.1
Hvu_XP_012567029.1
Hvu_XP_002166788.1
0.3

F

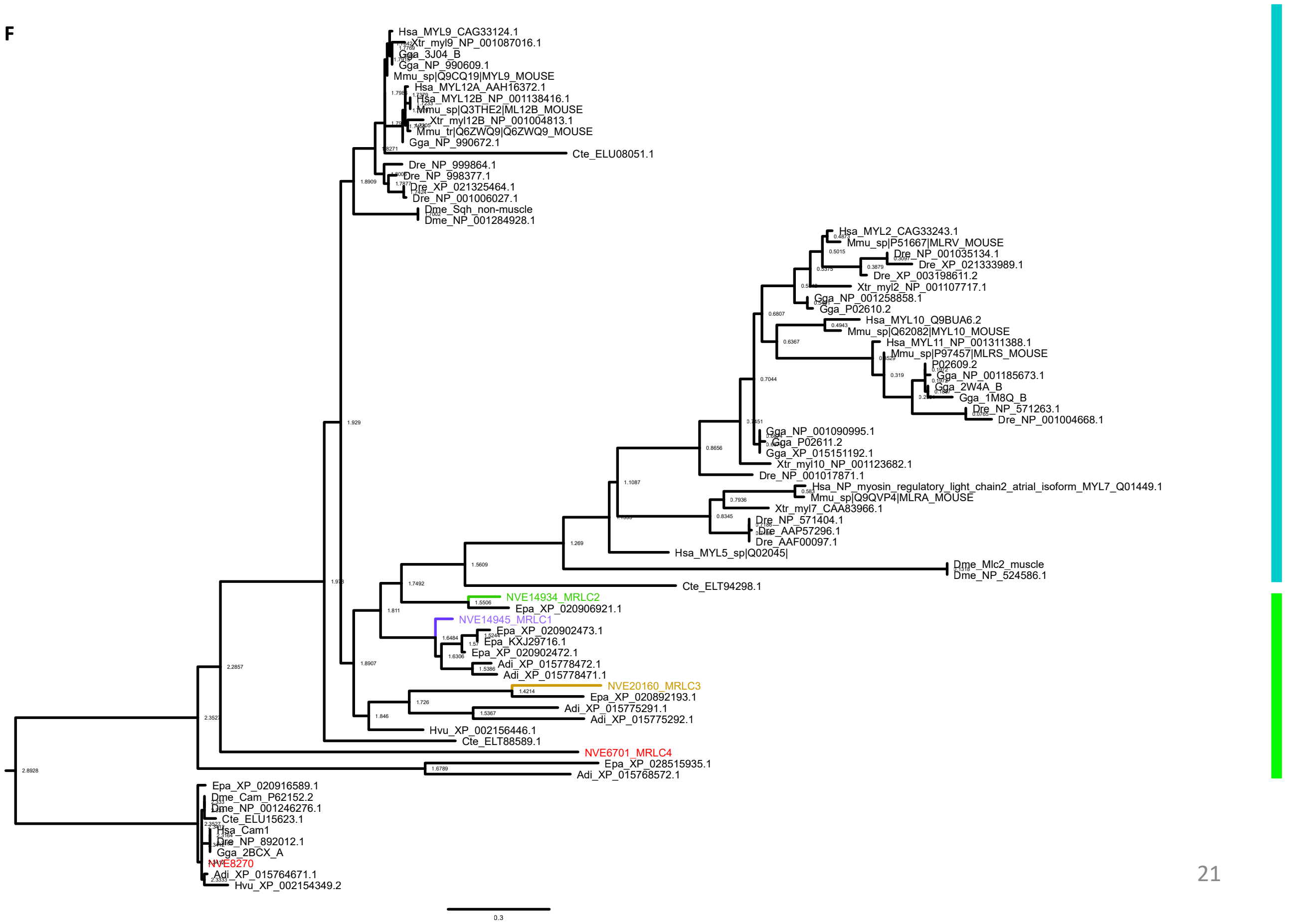

Hsa_MYL9_CAG33124.1
Xtr_myl9_NP_001087016.1
Gga_3J04_B
Gga_NP_990609.1
Mmu_sp|Q9CQ19|MYL9_MOUSE
Hsa_MYL12A_AAH16372.1
Hsa_MYL12B_NP_001138416.1
Mmu_sp|Q3THE2|ML12B_MOUSE
Xtr_myl12B_NP_001004813.1
Mmu_tr|Q6ZWQ9|Q6ZWQ9_MOUSE
Gga_NP_990672.1
Cte_ELU08051.1
Dre_NP_999864.1
Dre_NP_998377.1
Dre_XP_021325464.1
Dre_NP_001006027.1
Dme_Sqh_non-muscle
Dme_NP_001284928.1
Hsa_MYL2_CAG33243.1
Mmu_sp|P51667|MLRV_MOUSE
Dre_NP_001035134.1
Dre_XP_021333989.1
Dre_XP_003198611.2
Xtr_myl2_NP_001107717.1
Gga_NP_001258858.1
Gga_P02610.2
Hsa_MYL10_Q9BUA6.2
Mmu_sp|Q62082|MYL10_MOUSE
Hsa_MYL11_NP_001311388.1
Mmu_sp|P97457|MLRS_MOUSE
P02609.2
Gga_NP_001185673.1
Gga_2W4A_B
Gga_1M8Q_B
Dre_NP_571263.1
Dre_NP_001004668.1
Gga_NP_001090995.1
Gga_P02611.2
Gga_XP_015151192.1
Xtr_myl10_NP_001123682.1
Dre_NP_001017871.1
Hsa_NP_myosin_regulatory_light_chain2_atrial_isoform_MYL7_Q01449.1
Mmu_sp|Q9QVP4|MLRA_MOUSE
Xtr_myl7_CAA83966.1
Dre_NP_571404.1
Dre_AAP57296.1
Dre_AAF00097.1
Hsa_MYL5_sp|Q02045|
Dme_Mlc2_muscle
Dme_NP_524586.1
Cte_ELT94298.1
NVE14934_MRLC2
Epa_XP_020906921.1
NVE14945_MRLC1
Epa_XP_020902473.1
Epa_KXJ29716.1
Epa_XP_020902472.1
Adi_XP_015778472.1
Adi_XP_015778471.1
NVE20160_MRLC3
Epa_XP_020892193.1
Adi_XP_015775291.1
Adi_XP_015775292.1
Hvu_XP_002156446.1
Cte_ELT88589.1
NVE6701_MRLC4
Epa_XP_028515935.1
Adi_XP_015768572.1
Epa_XP_020916589.1
Dme_Cam_P62152.2
Dme_NP_001246276.1
Cte_ELU15623.1
Hsa_Cam1
Dre_NP_892012.1
Gga_2BCX_A
NVE8270
Adi_XP_015764671.1
Hvu_XP_002154349.2
0.3

Supplementary Figure 7

Illustration of image frame measurements for calculation of contraction rates as shown in figure 2D. The measured vector is indicated for all three measurement types: tentacle retraction (blue), retraction of the body column (red) as a proxy for the mesentery retractor, and body width as a proxy for peristaltic contraction (green). The distance used as circumferential contraction distance is calculated as indicated in **d**. See Supplementary Data2 for raw data, and Supplementary Movie1 for example image collection. Scale bars are 500μM.

a

Tentacle Retraction

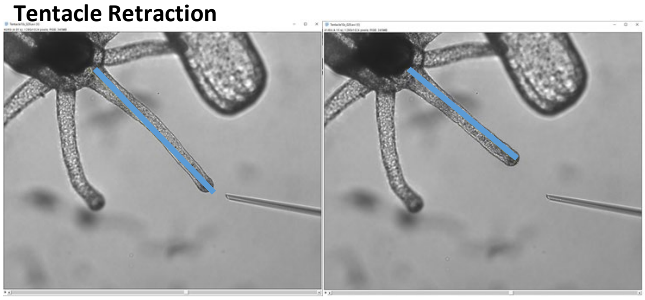

b

Peristaltic Contraction (CM)

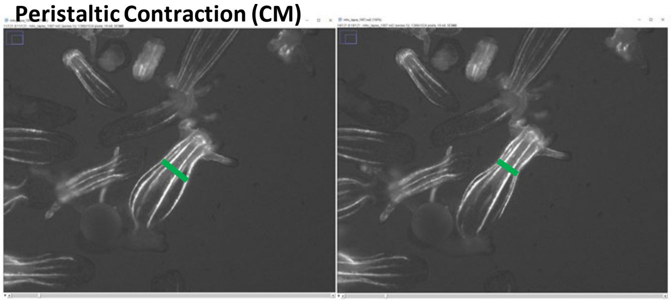

c **Body Retraction (mesentery retractor)**

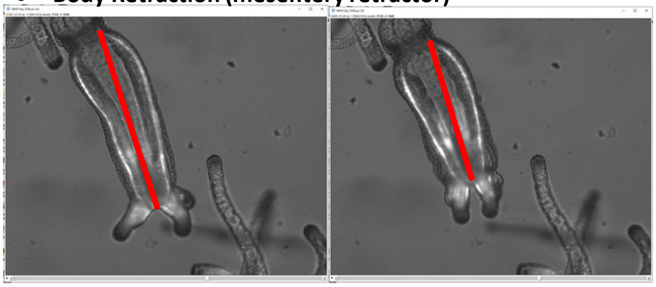

d

measuring the movement of two MHC filaments may yield in a measurement of only one direction of movement since again the animal is 3d but the spatial orientation of the filaments can not be discerned from this view

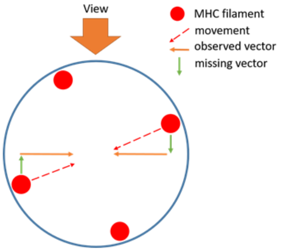

Supplementary Figure 8:

Expression profiles of transcription factors. **a** Differentially expressed transcription factors from the Muscle subset, as calculated with the Seurat 'FindMarkers' function, restricting the genes set to only those with DNA-binding motifs. Expression profiles of top ten significant genes are plotted onto the full dataset. **b** VennDiagram of overlapping genes of all detectable transcription factors (min. 5 reads) from each differentiated muscle cluster. **c** Expression profiles of genes from the set shown in B, filtered for having fewer than 25 reads within the ectodermal cell clusters of the full dataset. **d** Expression profiles of all putative orthologs of transcription factor families with known roles in muscle development in bilaterians. Putative members of the gene families were filtered for expression of at least 5 reads within any muscle cell population. **e** Expression of a metabotropic glutamate receptor specific to the tentacle retractor in the single cell dataset (left) and visualized with *in situ* hybridization (right). This receptor is specific to the tentacle retractor muscle (TR), and appears at 5 days post fertilization (dpf). **f** Double fluorescent in situ hybridization for nem64 (purple) and elaV (yellow) from the base of an adult tentacle. Co-localization in some cells of signal for both genes (arrows) can be seen in the enlarged images. See also Supplementary Data S1for full gene lists. Scale bars are 50μM.

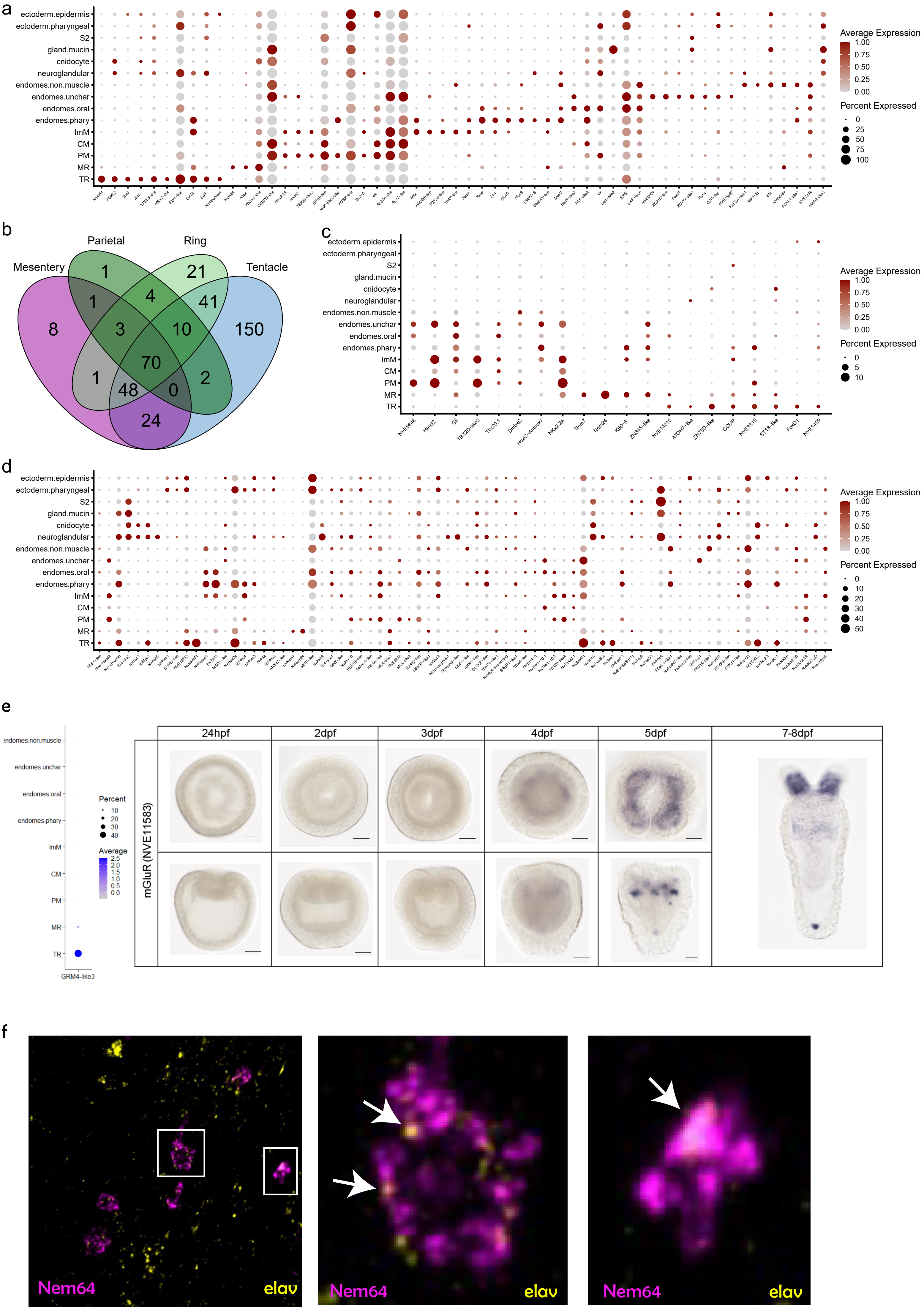
a
ectoderm.epidermis
ectoderm.pharyngeal
S2
gland.mucin
cnidocyte
neuroglandular
endomes.non.muscle
endomes.unchar
endomes.oral
endomes.phary
ImM
CM
PM
MR
TR
Average Expression
1.00
0.75
0.50
0.25
0.00
Percent Expressed
0
25
50
75
100
b
Mesentery
Parietal
Ring
Tentacle
8
1
21
150
1
4
41
3
10
70
1
48
0
2
24
c
Average Expression
Percent Expressed
0
5
10
NVE9848
Hand2
Gli
TBX20-like2
Tbx20.1
DmbxC
HoxC-Anthox7
NKx2.2A
Nem7
Nem24
K50-6
ZN345-like
NVE14215
ATOH7-like
ZN75D-like
COUP
NVE3315
ST18-like
FoxD1
NVE5459
d
Average Expression
Percent Expressed
0
10
20
30
40
50
e
endomes.non.muscle
endomes.unchar
endomes.oral
endomes.phary
ImM
CM
PM
MR
TR
GRM4-like3
Percent
10
20
30
40
Average
2.5
2.0
1.5
1.0
0.5
0.0
mGluR (NVE11583)
24hpf
2dpf
3dpf
4dpf
5dpf
7-8dpf
f
Nem64
elav
Nem64
elav
Nem64
elav

Supplementary Figure 9

a bHLH protein phylogenetic trees calculated with ngphylogeny.fr using the FastME/OneClick method, with 100 bootstrap calculation. Note that cnidarians do not have homologs of the MyoD-MRF family, but have significantly expanded independently (orange box). **b** Locus containing the cnidarian bHLH PaTH-family genes nem7, nem24, and nem64, as well as twist and paraxis orthologs. **c** Nk-family phylogenetic trees calculated with ngphylogeny.fr using the PhyML /OneClick method, with 100 bootstrap calculation. A Cnidarian-specific radiation of VND-related NK2.2 genes is evident (orange box). *Nematostella* sequences are highlighted in red.

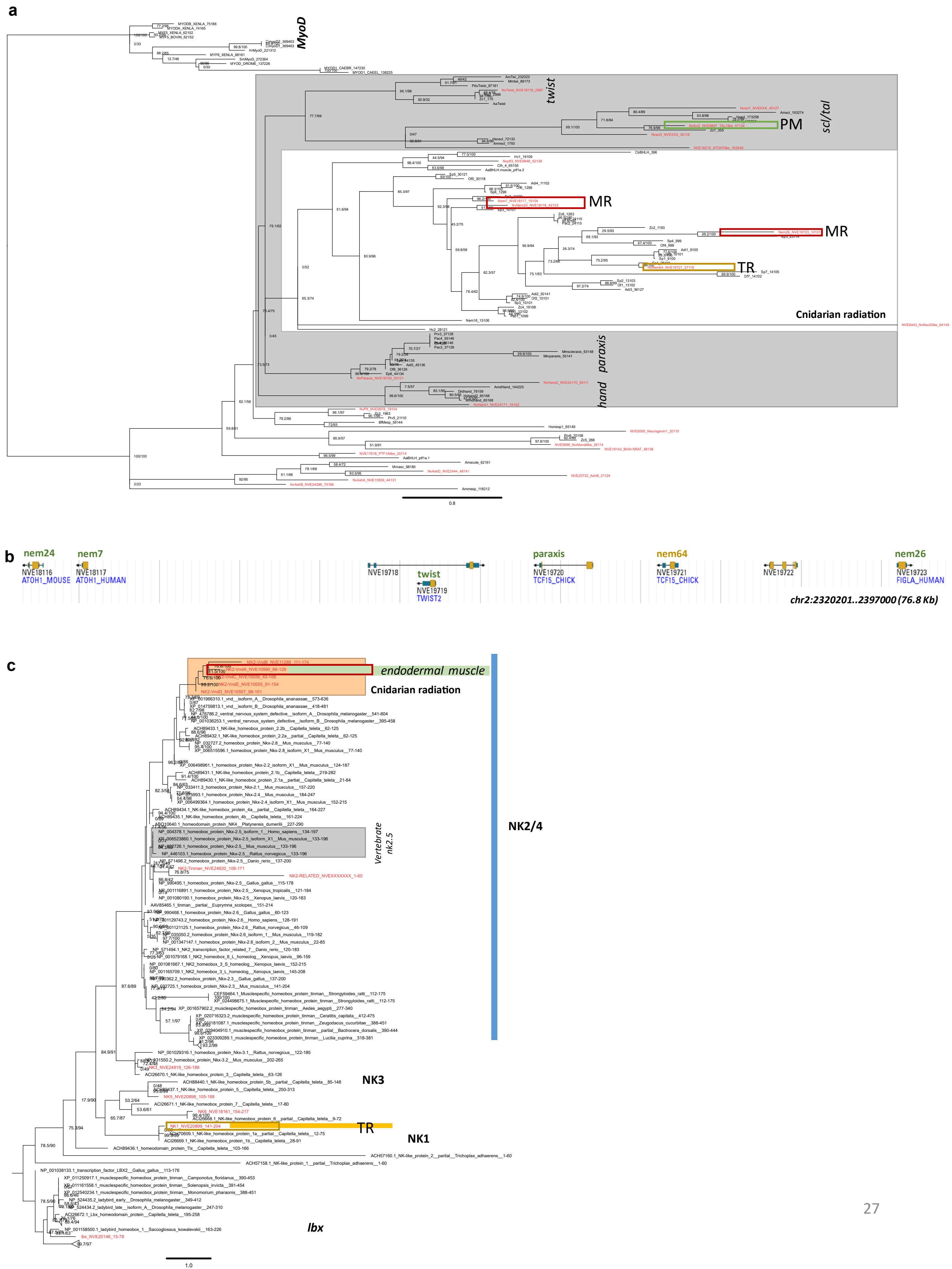
a
MyoD
twist
scl/tal
PM
MR
MR
TR
Cnidarian radiation
paraxis
hand
0.8
b
nem24
nem7
paraxis
nem64
nem26
NVE18116
ATOH1_MOUSE
NVE18117
ATOH1_HUMAN
NVE19718
twist
NVE19719
TWIST2
NVE19720
TCF15_CHICK
NVE19721
TCF15_CHICK
NVE19722
NVE19723
FIGLA_HUMAN
chr2:2320201..2397000 (76.8 Kb)
c
endodermal muscle
Cnidarian radiation
NK2/4
Vertebrate nk2.5
NK3
TR
NK1
lbx
1.0

Supplementary Figure 10

Schematic representation of the mutations introduced via CRISPR-cas9 for **a** E-protein, and **b** Nem64. In both cases a premature stop codon is introduced. **c** Expression profiles of identified muscle markers plotted across the two fast muscle cell types, separated by library. Nem64 and CALM-like3 are markers of the TR (left); MELC4 is expressed in both (center purple bar); NVE8057, Nem24, and DMBX1-like (right) are markers of MR; Note the absence of any TR markers in cells from the nem64 mutant library (bottom). TR: tentacle retractor (blue); MR: mesentery retractor (dark red).

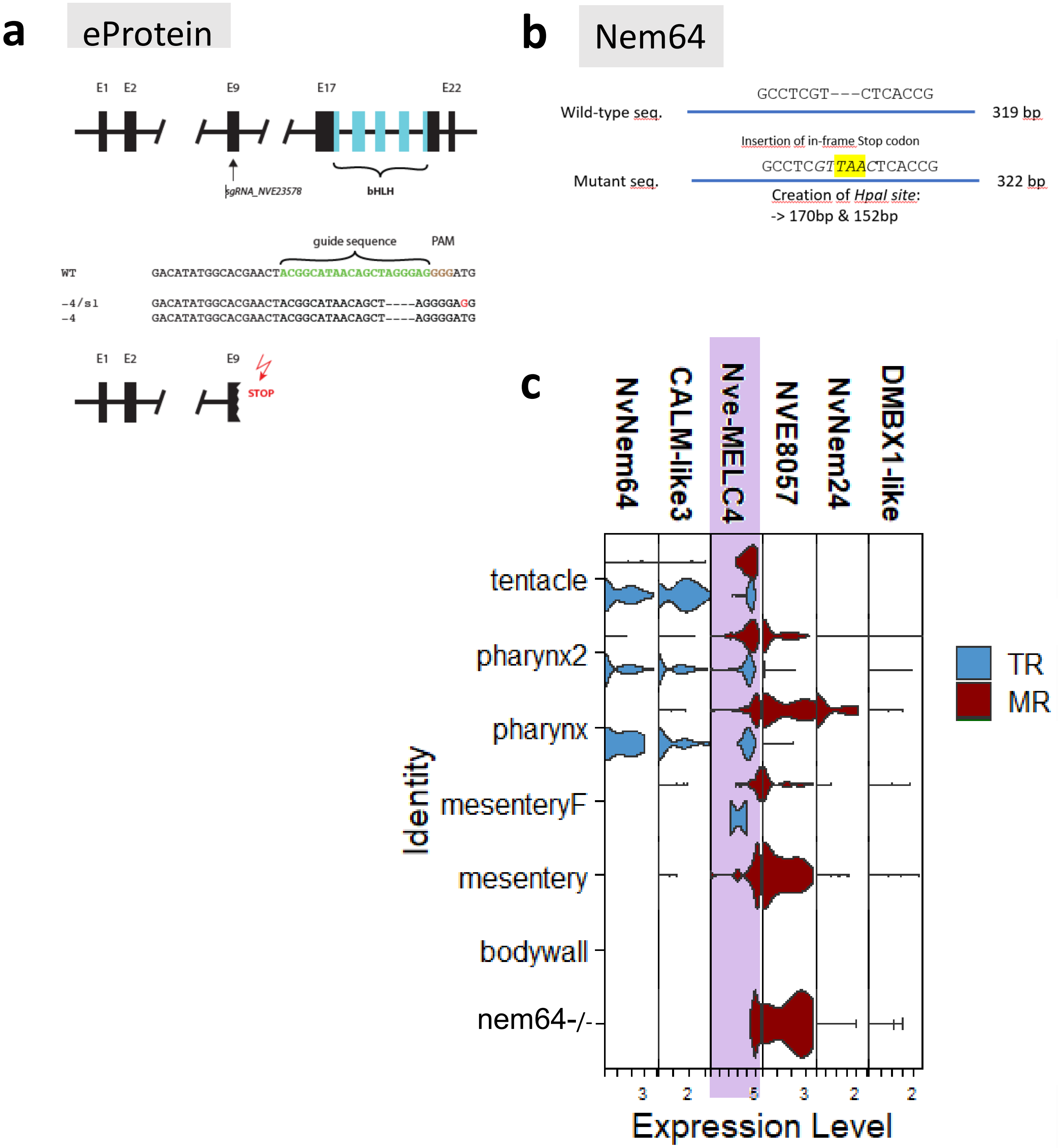
a
eProtein
E1 E2 E9 E17 E22
sgRNA_NVE23578
bHLH
guide sequence
PAM
WT GACATATGGCACGAACTACGGCATAACAGCTAGGGAGGGGATG
-4/s1 GACATATGGCACGAACTACGGCATAACAGCT----AGGGGAGG
-4 GACATATGGCACGAACTACGGCATAACAGCT----AGGGGATG
E1 E2 E9
STOP
b
Nem64
GCCTCGT---CTCACCG
Wild-type seq.
319 bp
Insertion of in-frame Stop codon
GCCTCGTTAACTCACCG
Mutant seq.
322 bp
Creation of HpaI site:
-> 170bp & 152bp
c
NvNem64
CALM-like3
Nve-MELC4
NVE8057
NvNem24
DMBX1-like
tentacle
pharynx2
pharynx
mesenteryF
mesentery
bodywall
nem64-/-
Identity
TR
MR
Expression Level
