# Supplementary material for: Muscle cell-type diversification is driven by bHLH transcription factor expansion and extensive effector gene duplications
Source: Nat Commun. 2023 Mar 29;14:1747. doi: 10.1038/s41467-023-37220-6 (PMC10060217; doi:10.1038/s41467-023-37220-6)
Supplement: Supplementary file 3 — Description of Additional Supplementary Files [file 41467_2023_37220_MOESM3_ESM.pdf]

Description of Additional Supplementary Files

Title: Supplementary Movie 1
Description: Example time-lapse film of MHC::mCherry transgenic line used to calculate contraction speeds as shown in Figure 2D. Measurements from single frames are found in Supplementary Data 2 . Scale bar is 100 µm.

Title: Supplementary Data 1
Description: Spreadsheets of gene lists derived from different analysis approaches. **Data 1.1** Cross reference between gene models and annotations. **Data 1.2**: list of upregulated genes in the BULK dataset shown in Figure 1c and S2c. **Data 1.3**: output of FindAllMarkers function of the Seurat Vs3 R package, for the clusters identified in Figure 1d for the full dataset. **Data 1.4**: output of FindAllMarkers function of the Seurat Vs3 R package, for the clusters identified in Figure 1e, excluding the set of all putative Transcription Factors. Expression profiles are imaged in Supplementary Figure 3e. **Data 1.5**: list of genes detected in any differentiated muscle cell population, excluding the set of all putative transcription factors, and absent from the ectodermal derivatives of the full dataset, as imaged in Supplementary Figure 5. **Data 1.6**: output of FindAllMarkers function of the Seurat Vs3 R package, for the clusters identified in Figure 1e from the set of all putative Transcription Factors. Expression profiles are imaged in Supplementary Figure 8a **Data 1.7**: list of all putative transcription factors detected (>5 reads) in any differentiated muscle cell population, but absent (<50 reads) from the ectodermal derivatives of the full dataset, as imaged in Figure Supplementary 8c. The intersection between the lists generated for each muscle cell type is illustrated in Supplementary Figure 8b. **Data 1.8** Gene sets used to annotate clusters in full Tissue dataset. **Data 1.9** Gene sets used to annotate clusters in Muscle data subset. **Data 1.10** Primer sequences used to generate in situ probes.

Title: Supplementary Data 2
Description: Spreadsheet of measurements used to calculate the muscle contraction speed shown in Figure 2d.

Title: Supplementary Data 3
Description: Sequence alignments used for generation of the phylogenetic trees in Supplementary Figures 7 & 10.
