# Supplementary material for: Muscle cell-type diversification is driven by bHLH transcription factor expansion and extensive effector gene duplications
Source: Nat Commun. 2023 Mar 29;14:1747. doi: 10.1038/s41467-023-37220-6 (PMC10060217; doi:10.1038/s41467-023-37220-6)
Supplement: Supplementary file 8 — Reporting Summary [file 41467_2023_37220_MOESM8_ESM.pdf]

nature portfolio

Corresponding author(s): Cole, Alison G. and Technau, Ulrich

Last updated by author(s): 03.01.2023

Reporting Summary

Nature Portfolio wishes to improve the reproducibility of the work that we publish. This form provides structure for consistency and transparency in reporting. For further information on Nature Portfolio policies, see our Editorial Policies and the Editorial Policy Checklist.

Statistics

For all statistical analyses, confirm that the following items are present in the figure legend, table legend, main text, or Methods section.

n/a	Confirmed	
☐	☒	The exact sample size (*n*) for each experimental group/condition, given as a discrete number and unit of measurement
☐	☒	A statement on whether measurements were taken from distinct samples or whether the same sample was measured repeatedly
☐	☒	The statistical test(s) used AND whether they are one- or two-sided<br>*Only common tests should be described solely by name; describe more complex techniques in the Methods section.*
☒	☐	A description of all covariates tested
☒	☐	A description of any assumptions or corrections, such as tests of normality and adjustment for multiple comparisons
☐	☒	A full description of the statistical parameters including central tendency (e.g. means) or other basic estimates (e.g. regression coefficient) AND variation (e.g. standard deviation) or associated estimates of uncertainty (e.g. confidence intervals)
☐	☒	For null hypothesis testing, the test statistic (e.g. *F*, *t*, *r*) with confidence intervals, effect sizes, degrees of freedom and *P* value noted<br>*Give P values as exact values whenever suitable.*
☒	☐	For Bayesian analysis, information on the choice of priors and Markov chain Monte Carlo settings
☒	☐	For hierarchical and complex designs, identification of the appropriate level for tests and full reporting of outcomes
☒	☐	Estimates of effect sizes (e.g. Cohen's *d*, Pearson's *r*), indicating how they were calculated

Our web collection on statistics for biologists contains articles on many of the points above.

Software and code

Policy information about availability of computer code

Data collection	Sequence mapping and count matrix generation: Cellranger 3.1.0
Data analysis	R 3.6.1, Seurat 3.1.4

For manuscripts utilizing custom algorithms or software that are central to the research but not yet described in published literature, software must be made available to editors and reviewers. We strongly encourage code deposition in a community repository (e.g. GitHub). See the Nature Portfolio guidelines for submitting code & software for further information.

Data

Policy information about availability of data

All manuscripts must include a data availability statement. This statement should provide the following information, where applicable:

- Accession codes, unique identifiers, or web links for publicly available datasets
- A description of any restrictions on data availability
- For clinical datasets or third party data, please ensure that the statement adheres to our policy

Raw sequence data generated in this study have been deposited in the GEO database under accession code GSE154477 [https://www.ncbi.nlm.nih.gov/geo/query/acc.cgi?acc=GSE154477]. The processed single cell data are available via the UCSC Cell Browser [sea-anemone-atlas.cells.ucsc.edu/]. The muscle contraction speed data generated in this study are provided as Supplementary Data 2. All other data is available in the main text and/or the Source Data file.

Human research participants

Policy information about studies involving human research participants and Sex and Gender in Research.

Reporting on sex and gender	NA
Population characteristics	NA
Recruitment	NA
Ethics oversight	NA

Note that full information on the approval of the study protocol must also be provided in the manuscript.

Field-specific reporting

Please select the one below that is the best fit for your research. If you are not sure, read the appropriate sections before making your selection.

☒ Life sciences ☐ Behavioural & social sciences ☐ Ecological, evolutionary & environmental sciences

For a reference copy of the document with all sections, see nature.com/documents/nr-reporting-summary-flat.pdf

Life sciences study design

All studies must disclose on these points even when the disclosure is negative.

Sample size	No sample size calculation was performed. A target of 3000 cells per each tissue library was selected for random capture via the 10x Genomics microfluidics platform.
Data exclusions	Sequenced droplets containing less than 200 expressed genes were excluded as non-informative. Droplets containing greater than 5000 molecules were excluded as potential doublets.
Replication	Tissue libraries of differing composition were generated from four separate individuals, separate time points, all yielding similar cell compositions.
Randomization	Cells from separate tissue dissociations are randomly captured via microfluidics.
Blinding	Study was not blind; assessment of origin of each cell transcriptome was necessary for both assessing data integration and biological meaning fulness of data analysis.

Reporting for specific materials, systems and methods

We require information from authors about some types of materials, experimental systems and methods used in many studies. Here, indicate whether each material, system or method listed is relevant to your study. If you are not sure if a list item applies to your research, read the appropriate section before selecting a response.

Materials & experimental systems

n/a	Involved in the study	
☒	☐	Antibodies
☒	☐	Eukaryotic cell lines
☒	☐	Palaeontology and archaeology
☐	☒	Animals and other organisms
☒	☐	Clinical data
☒	☐	Dual use research of concern

Methods

n/a	Involved in the study	
☒	☐	ChIP-seq
☒	☐	Flow cytometry
☒	☐	MRI-based neuroimaging

Animals and other research organisms

Policy information about studies involving animals; ARRIVE guidelines recommended for reporting animal research, and Sex and Gender in Research

Laboratory animals	Mature adults from a laboratory culture of Nematostella vectensis.

Wild animals	No wild animals were used
Reporting on sex	Sex information was not determined.
Field-collected samples	No samples were collected from the field
Ethics oversight	no ethical approval was required for use of cnidarian tissues.

Note that full information on the approval of the study protocol must also be provided in the manuscript.
